# Genetic outcomes in children with developmental language disorder: a systematic review

**DOI:** 10.3389/fped.2024.1315229

**Published:** 2024-01-17

**Authors:** Vivian van Wijngaarden, Hester de Wilde, Dieuwke Mink van der Molen, Jildo Petter, Inge Stegeman, Ellen Gerrits, Adriana L. Smit, Marie-José van den Boogaard

**Affiliations:** ^1^ Department of Genetics, University Medical Center Utrecht, Utrecht, Netherlands; ^2^ Department of Pediatric Otorhinolaryngology, Wilhelmina Children’s Hospital, University Medical Center Utrecht, Utrecht, Netherlands; ^3^ Faculty of Medicine, University Medical Center Utrecht, Utrecht University, Utrecht, Netherlands; ^4^ Department of Otorhinolaryngology and Head & Neck Surgery, University Medical Center Utrecht, Utrecht, Netherlands; ^5^ Brain Center, University Medical Center Utrecht, Utrecht, Netherlands; ^6^ Research Group Speech and Language Therapy, HU University of Applied Sciences Utrecht, Utrecht, Netherlands; ^7^ Department of Languages, Literature and Communication, Faculty of Humanities, Utrecht University, Utrecht, Netherlands

**Keywords:** children, developmental language disorder (DLD), diagnostic, genes, genetic etiology

## Abstract

**Introduction:**

Developmental language disorder (DLD) is a common childhood condition negatively influencing communication and psychosocial development. An increasing number of pathogenic variants or chromosomal anomalies possibly related to DLD have been identified. To provide a base for accurate clinical genetic diagnostic work-up for DLD patients, understanding the specific genetic background is crucial. This study aims to give a systematic literature overview of pathogenic variants or chromosomal anomalies causative for DLD in children.

**Methods:**

We conducted a systematic search in PubMed and Embase on available literature related to the genetic background of diagnosed DLD in children. Included papers were critically appraised before data extraction. An additional search in OMIM was performed to see if the described DLD genes are associated with a broader clinical spectrum.

**Results:**

The search resulted in 15,842 papers. After assessing eligibility, 47 studies remained, of which 25 studies related to sex chromosome aneuploidies and 15 papers concerned other chromosomal anomalies (SCAs) and/or Copy Number Variants (CNVs), including del15q13.1–13.3 and del16p11.2. The remaining 7 studies displayed a variety of gene variants. 45 (candidate) genes related to language development, including *FOXP2*, *GRIN2A*, *ERC1*, and *ATP2C2*. After an additional search in the OMIM database, 22 of these genes were associated with a genetic disorder with a broader clinical spectrum, including intellectual disability, epilepsy, and/or autism.

**Conclusion:**

Our study illustrates that DLD can be related to SCAs and specific CNV's. The reported (candidate) genes (*n* = 45) in the latter category reflect the genetic heterogeneity and support DLD without any comorbidities and syndromic language disorder have an overlapping genetic etiology.

## Introduction

A delayed or deviant language development is one of the most common disabilities in childhood (1). However, there has been a lack of agreement in literature and between practitioners about the exact criteria and terminology related to children's language problems.

The complex nature of language contributes to the challenges of identifying and classifying language impairments. Language involves understanding and using words and sentences to convey thoughts and information and should not be conflated with speech, which pertains to the production of vocal sounds.

In the context of scientific literature, there are challenges in precisely defining these concepts. Not only are different terms used to refer to language problems (e.g., specific language impairment, language delay, developmental verbal dyspraxia), at times terms referring to speech problems like “speech delay” are used to refer to problems in language. This lack of agreement about criteria and terminology for children's language problems hinders research and practice (2).

In their Delphi consensus study CATALISE of 2017, Bishop et al. (3, 4) aimed to reach international and multidisciplinary consensus in terminology regarding language impairment in children. Although the discussion about terminology and classification of language disorders continues to date, Bishop and colleagues propose the use of the term “Developmental Language Disorder” (DLD) to refer to cases of language disorder with no known differentiating conditions. These differentiating conditions include autism, epilepsy and intellectual disability. In the case of a language disorder co-occurring with one of these differentiating conditions, they recommend the use of the term “Language disorder (LD) associated with X”, where X is the differentiating condition. In their study, it was agreed that the presence of biological or environmental risk factors did not preclude a diagnosis of DLD, that DLD can co-occur with other neurodevelopmental disorders not related to language development and that DLD does not require a mismatch between language and nonverbal intelligence.

Although the debate on the terminology surrounding language issues remains unresolved, we adopt the terminology suggested by Bishop et al. for the sake of clarity. This means that in this article, we will use a strict definition of the term Developmental Language Disorder (DLD) for a language disorder with no known differentiating conditions.

Much is still unknown about the precise mechanisms behind language disorders. It is suggested that there may be multiple risk factors, which can reinforce each other, like chronic otitis media (5), socio-economic status (6), and oral-motor difficulties (7). As stated above, it is well known that language problems can co-occur with autism spectrum disorder (8), behavioral disorders (9) and certain epilepsy disorders (10). Furthermore, family and twin studies have indicated that DLD have a strong genetic component (11).

In the last decade, there has been an enormous progress in knowledge about the clinical features and genetic background of DLD, and conditions associated with DLD (12, 13). Furthermore, an increasing number of (candidate) genes possibly involved in the underlying mechanisms of DLD are reported. In particular, advances in molecular technologies are shedding new light on the genetic architecture underlying language-related disorders (14). Linkage and genome wide association studies (GWAS) resulted in several interesting genetic loci that seem to be associated with DLD and contribute to the risk of developing DLD with a multifactorial basis (15).

However, language disorders can also be Mendelian (or monogenic) in nature, which implies that the disorder involves a single genetic locus or gene. Mendelian disorders are caused by rare genetic variants with a large effect size. Several Mendelian causes for DLD have been identified over the years (13).

At this moment, it is not yet clear to what extent DLD is the result of monogenic causes and what part can be explained by multifactorial causes.

Mountford et al. (16) provided an overview of the genetic landscape of language disorders associated with a medical condition. However, in children with DLD, without any possibly explanatory comorbidities, a comprehensive overview of genes specifically related to DLD is missing. Such an overview could help in the diagnostic work-up of these patients. An early genetic diagnosis can be important for early treatment and better outcomes. Besides this, having an overview of genetic causes of DLD would be a good starting point for elaborating on the role of DLD within clinical genetic diagnostics, and it would provide a basis for further research regarding the combination of DLD and comorbidities like intellectual disability, epilepsy, and/or autism spectrum disorder. Gaining a deeper understanding of the genetic origins of DLD would offer valuable theoretical insights into the connection between genes, brain functionality, cognition, and particularly the language faculty.

With this review, we aim to give an overview of the literature in children on pathogenic variations or chromosomal anomalies related to DLD.

## Methods

This review is reported according to Preferred Reporting Items for Systematic Review and Meta-Analysis PRISMA-statement (17).

### Aim

Which specific pathogenic variants and chromosomal anomalies are reported in the literature to be related to developmental language disorder in children?

### Inclusion criteria

In the present review study, we used the term “developmental language disorder” or “DLD” as an umbrella term for the different fields in which language problems can occur: phonology, (morpho)syntax, semantics and pragmatics. We used a strict definition of “developmental language disorder” or “DLD”, to refer to language problems in the absence of other neurodevelopmental disorders or other confounding issues.

Inclusion criteria included original research describing children with developmental language disorder associated with a specific genetic variation written in English or Dutch for which a full text was available. Inclusion was based on the existence of problems in language development in described cases, resulting from language tests or because this was stated by the authors, in the absence of any known confounding variables.

### Exclusion criteria

Reviews, linkage studies, genetic association studies, articles based on animal models, articles in which insufficient details were given regarding the genetic variant, and articles in which the subjects were adults or had hearing problems, clefts, dyslexia, anatomical issues that could affect speech (e.g., velopharyngeal insufficiency (VPI), or when the speech language problems solely consisted of motor speech problems, and not language problems, were excluded. Furthermore, articles in which the language problems were not isolated but patients presented with comorbidities like intellectual disability (IQ <70), epilepsy/seizures, autism spectrum disorder (ASD) and/or ataxia were excluded.

### Search strategy

We searched the PubMed- and Embase-databases for studies on the genetics of DLD. All articles published until April 1, 2023 were included. An overview of the search queries used, both for PubMed and Embase, can be found in Appendix 1.

### Study selection

The articles resulting from the search query in Embase and PubMed were deduplicated by two reviewers (VW and HW) and screened independently by four reviewers (VW, HW, DM and JP) using title and abstract on relevance to the topic of interest. Articles not related to the topic were excluded. Reviewer 1 (VW) is a linguist/speech, language, and hearing researcher; reviewer 2 (HW) is a linguist/speech language pathologist; reviewer 3 (DM) and 4 (JP) are medical students. The full text of the screened articles was assessed independently by two reviewers (VW and DM). Only articles that met the inclusion and exclusion criteria were included for qualitative analysis. Conflicts were resolved by discussion and in the case of disagreement, a third researcher weighed in.

### Risk of bias assessment

The articles included in the review were critically appraised using the relevant Critical Appraisal Tools (CAT) of the Joanna Briggs Institute for the relevant study design. The papers regarding sex chromosome aneuploidies were assessed independently by reviewer VW and reviewer JP. The papers regarding other pathogenic variants were assessed independently by reviewer VW and reviewer HW.

As overall consideration for possible bias were considered a clear description and demographic information of the patients and appropriate identification and measurement of the condition and valid statistical analysis. Furthermore, in cases series, clear criteria for inclusion and complete and consecutive inclusion were considered. In case control studies, it was considered if both the group of patients in which the condition was present and the group in which the condition was absent were otherwise comparable. In prevalence studies, it was considered if the sampling was appropriate.

Since intervention procedures, treatment and exposure parameters are not relevant for the topic of interest of this review, we have not scored these checklist items in the study designs case reports (checklist items 4–6 regarding intervention and treatment), case control studies (checklist items 4, 5 and 9 regarding exposure) and case series (checklist item 8).

### Data extraction and synthesis

Relevant data including the study characteristics (first author's name, year of publication), study design, subject information (number of subjects, age), genetic background (type of mutation, genetic tests), linguistic background (language test, language profile) were extracted from the studies included in the qualitative review. Data extraction was done by VW and HW for double checking.

In the synthesis, reported genetic backgrounds of the subjects described in the included studies were described. Subsequently, an descriptive analysis was performed for interpretation of the found mutations. The found genes were checked in OMIM, to see if they were known OMIM morbid genes related to a genetic disorder. An additional search was performed in PubMed to reveal any possible relation between described genetic variants and intellectual disability, epilepsy, autism, and/or dyslexia. Outcomes of genetic findings on different subgroups were described, including clinical spectrum of reported genes and the relation with linguistic background of reported children.

## Results

### Study selection

**Figure 1:**
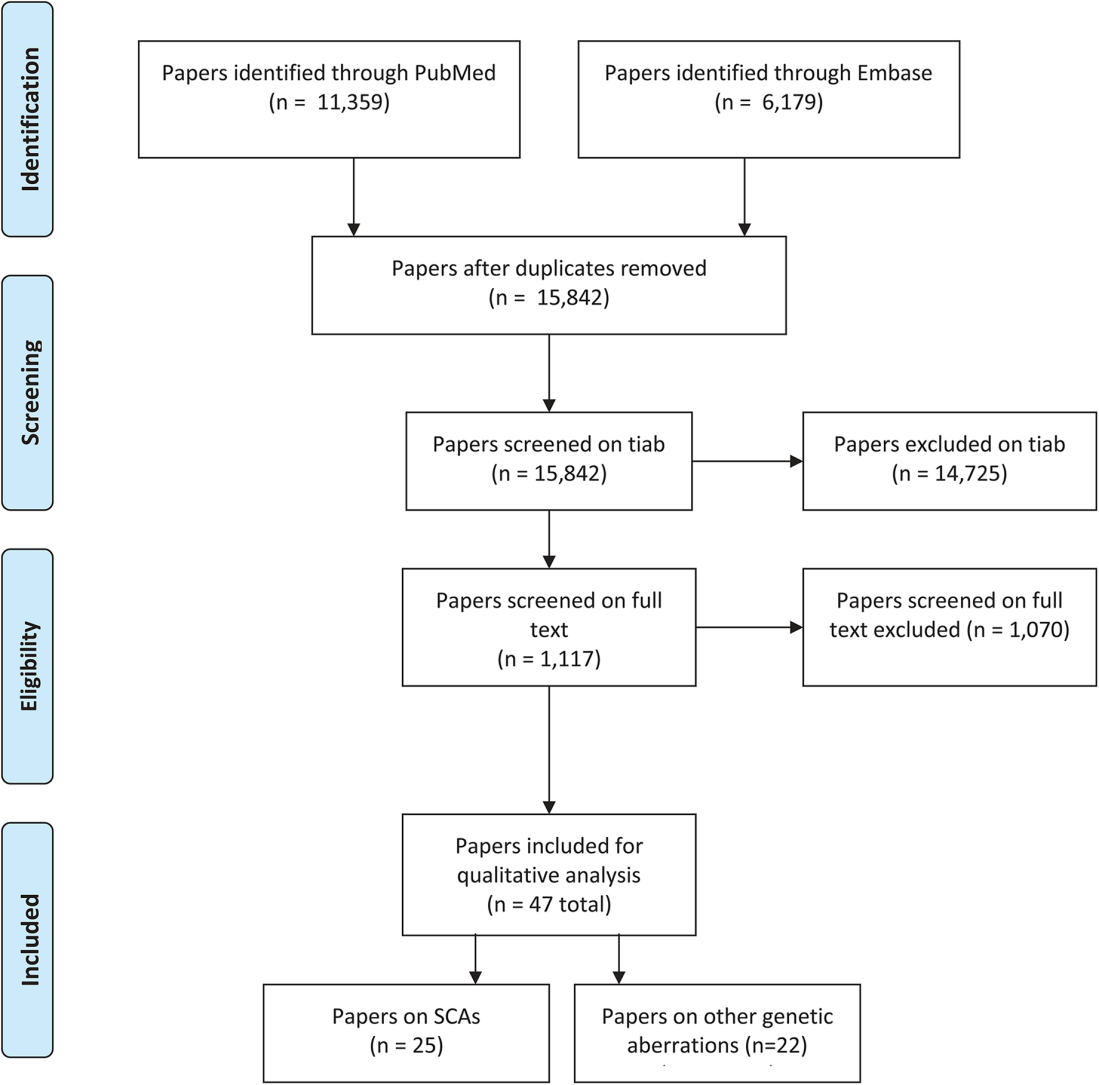
PRISMA-flowchart screening.

The search query in Embase and PubMed resulted in 15,842 studies after deduplication. After screening on title and abstract, a total of 14,725 were excluded and 1,117 were reviewed on full text. This screening on full text resulted in 47 studies included for critical appraisal (see Figure 1).

The vast majority of papers (11,039) were excluded because of the exclusion criteria linkage studies, genetic association studies, articles in a language other than English or Dutch, articles based on animal models, articles in which insufficient details were given regarding the genetic mutation, articles in which the subjects had ataxia, motor speech problems, hearing problems, clefts, dyslexia, anatomical issues that could affect speech (e.g., velopharyngeal insufficiency (VPI) or adult subjects or the article completely lacked relevance to the topic of interest. Relatively many papers (*n* = 3,686) were excluded because of the combination of DLD with intellectual disability (IQ <70) (*n* = 2,727), epilepsy/seizures (*n* = 1,237), and/or autism spectrum disorder (*n* = 888). (Please note that papers can be excluded because of multiple exclusion criteria, e.g., both ID and epilepsy, see Table 1).

**Table 1:** Papers excluded based on exclusion criteria ID, epilepsy, and/or autism.

Total number of papers after initial search query	15,842
Total number of papers excluded after first screening on title and abstract	14,725
Total number of papers excluded for criteria ID, epilepsy and/or ASD	3,686
Number of papers excluded based on:^a^	
ID (IQ < 70)	2,727
Epilepsy/seizures	1,237
Autism Spectrum Disorder (ASD)	888
Number of papers excluded for other exclusion criteria^b^	11,039
Total number of papers included in review	47

^a^ Papers can be excluded because of multiple exclusion criteria, e.g., both ID and epilepsy.

^b^ Reviews, linkage studies, genetic association studies, articles in a language other than English or Dutch, articles based on animal models, articles in which insufficient details were given regarding the genetic mutation, articles in which the subjects had ataxia, motor speech problems, hearing problems, clefts, dyslexia, anatomical issues that could affect speech (e.g., velopharyngeal insufficiency (VPI), adult subjects.

### Study characteristics of included papers

A total of 47 papers were finally included describing children with developmental language disorders in relation to a specific pathogenic variants or chromosomal anomalies, without any other possibly explanatory risk factor. Concerning the study design, 18 papers were case control studies, 14 papers were case reports, 9 papers were case series and 6 papers were prevalence studies. Studies were published between 1973 and 2023.

### Results risk of bias assessment

Of the 47 studies included, 34 studies have met 80%–100% of the JBI-criteria and were classified as low risk of bias, 9 studies met 60%–80% of the JBI-criteria and were classified as moderate risk of bias, and 4 studies met less than 60% of the JBI-criteria and were therefore classified as high risk of bias.

There were 14 case reports: 1 paper with a high risk of bias, 1 with a moderate risk of bias and 12 with a low risk of bias. The moderate and high risk of bias was mainly due to insufficient description of the condition and the fact that diagnostic tests/assessments were not clearly described.

9 papers were case series: 1 paper had a high risk of bias, 5 a moderate risk of bias and 3 a low risk of bias. The high and moderate risk of bias was due to the fact that it was not clear if inclusion of participants was complete and consecutive and because of unclear reporting of the demographic and/or clinical information of the participants.

Of the 18 case control studies, 1 had a high risk of bias, 3 a moderate risk of bias and 14 a low risk of bias. The high and moderate risk of bias was mainly because it was unclear if the groups were appropriately matched and because confounding factors were not mentioned or dealt with appropriately.

Finally, there were 6 prevalence studies. 1 of these papers had a high risk of bias, the other 5 papers had a low risk of bias. In the study with the high risk of bias, it was unclear if the sampling was done appropriately.

The results of the critical appraisal of each individual study are depicted in Tables 2A–D.

**Table 2A:** Critical appraisal of papers: CAT case control.

Author	Year of publication	1. Groups comparable?	2. Cases and controls matched appropriately?	3. Same criteria for identification?	4. Exposure measured in valid and reliable way?	5. Exposure measured in same way for cases/controls?	6. Confounding factors?	7. Strategies to deal with confounding factors?	8. Outcomes assessed in valid and reliable way?	9. Exposure period of interest long enough?	10. Appropriate statistical analysis?	Score	Risk of bias
Addis	2010	Yes	Yes	Yes	n/a	n/a	No	No	Yes	n/a	Yes	5	Moderate
Andres	2021	Yes	Yes	Yes	n/a	n/a	Yes	Yes	Yes	n/a	Yes	7	Low
Bender	1983	Yes	Yes	Yes	n/a	n/a	Yes	Yes	Yes	n/a	Yes	7	Low
Bishop	2011	Yes	Unclear	Yes	n/a	n/a	Yes	Yes	Yes	n/a	Yes	6 or 7	Low
Bishop	2019	Yes	Yes	Yes	n/a	n/a	Yes	Yes	Yes	n/a	Yes	7	Low
Bishop & Scerif	2011	Yes	Unclear	Yes	n/a	n/a	Yes	Yes	Yes	n/a	Yes	6 or 7	Low
IJst	2002	Unclear	Unclear	Unclear	n/a	n/a	No	No	No	n/a	No	0–3	High
Kalnak	2018	Yes	Yes	Yes	n/a	n/a	Yes	Yes	Yes	n/a	Yes	7	Low
Lee	2012	Yes	Yes	Yes	n/a	n/a	Yes	Yes	Yes	n/a	Yes	7	Low
Matsuzaki	2019	Yes	Yes	Yes	n/a	n/a	Yes	Yes	Yes	n/a	Yes	7	Low
Rakonjac	2015	Yes	Yes	Yes	n/a	n/a	No	No	Yes	n/a	Yes	5	Moderate
Ross	2009	Yes	Yes	Yes	n/a	n/a	Yes	Yes	Yes	n/a	Yes	7	*Low*
Rovet	1996	Yes	Yes	Yes	n/a	n/a	Yes	Yes	Yes	n/a	Yes	7	*Low*
Udhnani	2018	Yes	Yes	Yes	n/a	n/a	Yes	Yes	Yes	n/a	Yes	7	Low
Urbanus	2022	Yes	Yes	Yes	n/a	n/a	Yes	Yes	Yes	n/a	Yes	7	Low
Urbanus	2023	Yes	Yes	No	n/a	n/a	Yes	Yes	Yes	n/a	Yes	6	Moderate
Zampini	2018	Yes	Yes	Yes	n/a	n/a	Yes	Yes	Yes	n/a	Yes	7	Low
Zampini	2022	Yes	Yes	Yes	n/a	n/a	Yes	Yes	Yes	n/a	Yes	7	Low

Full description:

1. Were the groups comparable other than the presence of disease in cases or the absence of disease in controls?

2. Were cases and controls matched appropriately?

3. Were the same criteria used for identification of cases and controls?

4. Was exposure measured in a standard, valid and reliable way?

5. Was exposure measured in the same way for cases and controls?

6. Were confounding factors identified?

7. Were strategies to deal with confounding factors stated?

8. Were outcomes assessed in a standard, valid and reliable way for cases and controls?

9. Was the exposure period of interest long enough to be meaningful?

10. Was appropriate statistical analysis used?

**Table 2B:** Critical appraisal of papers: CAT case reports.

Author	Year of publication	1. Patient's demographic characteristics	Patient's history clearly described?	3. Current clinical condition clearly described?	4. Diagnostic tests/assessment methods and results clearly described?	*5. Intervention(s)/treatment procedures clearly described?*	*6. Post-intervention clinical condition clearly described?*	*7. Adverse events/unanticipated events identified and described?*	8. Takeaway lessons?	Score	Risk of bias
Bogart	1986	Yes	Yes	Yes	No	*n/a*	*n/a*	*n/a*	Yes	4	Low
Ceroni	2014	Yes	Yes	Yes	No	*n/a*	*n/a*	*n/a*	Yes	4	Low
Chui	2011	Yes	Yes	Yes	Yes	*n/a*	*n/a*	*n/a*	Yes	5	Low
Kwasnicka	2005	Yes	Yes	Yes	Yes	*n/a*	*n/a*	*n/a*	Yes	5	Low
Lai	2000	Yes	Yes	Yes	No	*n/a*	*n/a*	*n/a*	Yes	4	Low
Lai^a^	2001	No	No	Yes	No	*n/a*	*n/a*	*n/a*	Yes	2	High
Moralli	2015	Yes	Yes	Yes	Yes	*n/a*	*n/a*	*n/a*	Yes	5	Low
Riccardi	2015	Yes	Yes	Yes	No	*n/a*	*n/a*	*n/a*	Yes	4	Low
Tomblin	2009	No	No	Yes	Yes	*n/a*	*n/a*	*n/a*	Yes	3	Moderate
Unger	2007	Yes	Yes	Yes	No	*n/a*	*n/a*	*n/a*	Yes	4	Low
Van Elst	2020	Yes	Yes	Yes	Yes	*n/a*	*n/a*	*n/a*	Yes	5	Low
Weimer	2006	Yes	Yes	Yes	Yes	*n/a*	*n/a*	*n/a*	Yes	5	Low
Weistuch	1996	Yes	Yes	Yes	Yes	*n/a*	*n/a*	*n/a*	Yes	5	Low
Yeung	2013	Yes	Yes	Yes	No	*n/a*	*n/a*	*n/a*	Yes	4	Low

Full description JBI Critical Appraisal Checklist for Case Reports:

1. Were patient's demographic characteristics clearly described?

2. Was the patient's history clearly described and presented as a timeline?

3. Was the current clinical condition of the patient on presentation clearly described?

4. Were diagnostic tests or assessment methods and the results clearly described?

5. Was the intervention(s) or treatment procedure(s) clearly described?

6. Was the post-intervention clinical condition clearly described?

7. Were adverse events (harms) or unanticipated events identified and described?

8. Does the case report provide takeaway lessons?

^a^ CS is same patient as in article 2,000.

**Table 2C:** Critical appraisal of papers: CAT case series.

Author	Year of publication	1. Clear inclusion criteria?	2. Condition measured in a standard, reliable way for all participants?	3. Valid methods used for identification of the condition?	4. Consecutive inclusion of participants?	5. Complete inclusion of participants?	6. Clear reporting of the demographics of the participants?	7. Clear reporting of clinical information of participants?	*8. Outcomes or follow up results of cases clearly reported?*	9. Clear reporting of the presenting site(s)/clinic(s) demographic information?	10. Statistical analysis appropriate?	Score	Risk of bias
Akcan	2018	Yes	Yes	Yes	Unclear	Unclear	Yes	No	*n/a*	Yes	yes	6 to 8	Moderate
Capelli	2022	Yes	Yes	Yes	Unclear	Unclear	Yes	Yes	*n/a*	Yes	yes	7 to 9	Low
Centanni	2015	Yes	Yes	Yes	No	No	Yes	Yes	*n/a*	No	yes	6	Moderate
Haka-Ikse	1978	Yes	Yes	Yes	Yes	No	Yes	Yes	*n/a*	No	yes	7	Low
LeGoff	2013	Yes	Unclear	Unclear	Unclear	Unclear	Yes	Yes	*n/a*	N/a	n/a	3 to 7	High
Pettigrew	2015	Yes	Yes	Yes	Yes	Unclear	Yes	Yes	*n/a*	Yes	yes	8 to 9	Low
Samango	2021	Yes	Unclear	Yes	Unclear	Unclear	Yes	Yes	*n/a*	Yes	yes	6 to 9	Moderate
Tennes	1977	Yes	Yes	Yes	No	No	Yes	Yes	*n/a*	Yes	n/a	6 to 7	Moderate
Thompson	1986	Yes	Yes	Yes	Unclear	Unclear	Yes	Yes	*n/a*	No	n/a	5 to 7	Moderate

Full description JBI Critical Appraisal Checklist for Case Series:

1. Were there clear criteria for inclusion in the case series?

2. Was the condition measured in a standard, reliable way for all participants included in the case series?

3. Were valid methods used for identification of the condition for all participants included in the case series?

4. Did the case series have consecutive inclusion of participants?

5. Did the case series have complete inclusion of participants?

6. Was there clear reporting of the demographics of the participants in the study?

7. Was there clear reporting of clinical information of the participants?

8. Were the outcomes or follow up results of cases clearly reported?

9. Was there clear reporting of the presenting site(s)/clinic(s) demographic information?

10. Was statistical analysis appropriate?

**Table 2D:** Critical appraisal of papers: prevalence.

Author	Year of publication	1. Sample frame appropriate for target population?	2. Study participants sampled in appropriate way?	3. Sample size adequate?	4. Study subjects and setting described in detail?	5. Data analysis with sufficient coverage of identified sample?	6. Valid methods for identification of condition?	7. Condition measured in standard, reliable way?	8. Appropriate statistical analysis?	9. Adequate response rate/low response rate manage appropriately?	Score	Risk of bias
Chen	2017	Yes	Yes	Yes	Yes	Yes	Yes	Yes	Yes	n/a	9	Low
Garvey	1973	No	No	No	Yes	n/a	Yes	No	Unclear	n/a	2 to 3	High
Gropman	2020	Yes	Yes	Yes	Yes	Unclear	Yes	Yes	Yes	n/a	7 to 8	Low
Ross	2008	Yes	Yes	Yes	Yes	n/a	Yes	Yes	Yes	n/a	9	Low
Simpson	2014	Yes	Yes	Yes	Yes	Yes	Yes	Unclear	Yes	Yes	8 to 9	Low
Stemkens	2006	Yes	Yes	Yes	Yes	Yes	Yes	Unclear	Yes	Yes	8 to 9	Low

Full description JBI Critical Appraisal Checklist for Studies Reporting Prevalence Data:

1. Was the sample frame appropriate to address the target population?

2. Were study participants sampled in an appropriate way?

3. Was the sample size adequate?

4. Were the study subjects and the setting described in detail?

5. Was the data analysis conducted with sufficient coverage of the identified sample?

6. Were valid methods used for the identification of the condition?

7. Was the condition measured in a standard, reliable way for all participants?

8. Was statistical analysis appropriate?

9. Was the response rate adequate, and if not, was the low response rate managed appropriately?

**Table:** 

	Low risk of bias	Moderate risk of bias	High risk of bias	Total number of papers
Case Control	14	3	1	18
Case Report	12	1	1	14
Case Series	3	5	1	9
Prevalence	5	0	1	6
	34	9	4	47

### Characteristics of the studied population

Of the 47 included studies, 25 papers reported on sex chromosome aneuploidies (SCAs). The remaining 22 studies discuss a variety of structural chromosomal anomalies (7 studies), Copy Number Variants (CNVs) (7 studies) and gene variants (7 studies) (Supplementary Tables 3A,B).

Data of study characteristics, study design, subject information (number of subjects, age), genetic background (type of mutation, genetic tests), and linguistic background (language test, language profile) of cases out of included papers can be found in Supplementary Table S5.

In the following section, we will elaborate on different subgroups of genetic findings, clinical spectrum of reported genes and the relation with linguistic background as reported in included studies.

#### Sex chromosomes aneuploidies (SCAs)

25 out of 47 included studies reported on cases with different sex chromosomes aneuplodies (see Table 3A): (18–42).

**Table 3:** Found SCAs and other chromosomal anomalies or pathogenic variants.

Author	Year	SCA (*n* =)
(A)		
Akcan	2018	47, XXY (23)
Bender	1983	47, XXY (14); 47, XYY (4); 47, XXX (9); 45, X (8), mosaics female (6)
Bishop	2011	47, XXY (19); 47, XYY (58); 47, XXX (58)
Bishop	2019	47, XXY (31); 47, XYY (31); 47, XXX (35)
Bishop & Scerif	2011	47, XXY (19); 47, XYY (21); 47, XXX (28)
Capelli	2022	47, XYY (6); 47 XXY (5); 47, XXX (2)
Garvey	1973	48, XXYY (1); mosaics (2)
Gropman	2020	49, XXXXY (67)
Haka-Ilse	1978	47, XXY (25); XYY (3); XXX (10); XX male (1), XXYY (1); XX/XO (1); XY/XXY (1)
Lee	2012	47, XXY (27); XYY (15); XXX (28); XXXX (3); XXXXX (1); XXXY (8); XXXXY (12)
Matsuzaki	2019	47, XYY (31)
Ross	2008	47, XXY (50)
Ross	2009	47, XXY (93); 47, XYY (21)
Rovet	1996	47, XXY (36)
Samango-Sprouse	2021	49, XXXXXY (85)
Simpson	2014	47, XXY (3); 47, XYY (3); 47,XXX (3); 47, XXY of XO/XY (1)
Stemkens	2006	47, XXY (61)
Tennes	1977	47, XXY (12); 47, XYY (4); 46, XY/XXY mosaicism (1)
Udhnani	2018	47, XXY (19); 47, XYY (11); 47, XXX (27); 48, XXXX (1); 48, XXXY (4); 48, XXYY (10) 49, XXXXY (7)
Urbanus	2022	47, XXX (32); 47, XXY (49); 47, XYY (22)
Urbanus	2023	47, XXX (27); 47, XXY (29); 47, XYY (16)
Van Elst	2020	47, XXX (1)
Weimer	2006	ring Y-chromosome and partial trisomy 8 (1)
Zampini	2018	47, XXY
Zampini	2022	47, XXX (14); 47, XXY (12); 47, XYY (12)


Author	Year	Mutation/Gene/Locus/Region (*n*=)	OMIM morbid gene
(B)			
Addis	2010	Locus maps to 12p13.31-q14.3 (*n* = 8, all members of same family); six candidate genes CNTN1, FOXJ2, GRIN2B, NAB2, NELL2 and SRGAP1 sequenced; variants detected in SRGAP1 and CNTN1	CNTN1:? Myopathy, congenital, Compton-North # 612540; GRIN2B: Developmental and epileptic encephalopathy; Developmental and epileptic encephalopathy 27 616139 AD 3 Intellectual developmental disorder, autosomal dominant 6, with or without seizures 613970
Andres	2022	Whole-exome sequencing (WES) in a single family (*n* = 11) identified co-segregating rare variants in three genes: *BUD13*, *APLP2*, and *NDRG2*. To determine the significance of these genes in SLI, all coding regions of each gene in unrelated individuals with SLI (*n* = 175) were sequenced. Variants in *BUD13* reached genome-wide significance (*p*-value < 0.01), providing gene level evidence that *BUD13* is involved in SLI. Additionally, five BUD13 variants showed cohesive variant level evidence of likely pathogenicity.	
Bogart	1986	46,XX,t(6;11)(p21;q21),t(11;21) (q21; pl3),inv(6)(p21qI 1) *de novo* (*n* = 1)	
Centanni	2015	Gains in 15q11.2 region (SNORD109A, SNORD109B and SNORD116) (*n* = 4), with additional CNV's (child 1: 13q21.1 and 12p13.33; child 2: 12p13.33 and 16p11.2; child 3: 9p24.3 and 22q13.33; child 4: 7q11.23)	15q11-q13 region is also implicated in Angelman syndrome and Prader-Willi syndrome
Ceroni	2014	Homozygous microdeletion involving exon 5 of ZNF277 (NM_021994.2) (chr7:g111955948_111960100del, hg19) (*n* = 1) The microdeletion falls within the AUTS1 locus, a region linked to autistic spectrum disorders (ASDs). Moreover, ZNF277 is adjacent to the DOCK4 and IMMP2l genes, which have been implicated in ASD. (Deletion was heterozygous in parents (both with language problems in childhood. Deletion was absent in brother with mild language impairment.)	AUTS1: {Autism susceptibility 1} OMIM % 209850
Chen	2017	Genevariants identified by Whole Exome Sequencing (*n* = 43): ATP2C2 (6); AUTS2 (2, 1 co-segregates in family); CNTNAP2 (1); CNTNAP5 (2); DCDC2 (3); ERC1 (3 total; 1 novel variant); GRIN2A (1, novel *de novo*); GRIN2B (1, novel); KIAA0319 (6); NFXL1 (1); ROBO1 (2); SEMA6D (3); SETBP1 (5); SRPX2 (1); // Stop-gain variants identified in SLIC probands: OR6P1 (1); SYNPR (1); OXR1 (1, co-segregates in family)); IDO2 (1); MUC6 (1, co-segregates in family); OR52B2 (1, in addition to variants in AUTS2, OR52B2, KIAA0586, STARD9); NUDT16L1 (1)// Genes with more than one rare variant in the same SLIC proband: SCN9A (1), BIRC6 (2), FLNB (1), OR52B2 (1), FAT3 (1), KMTD2 (1), MYO16 (1), KIAA0586 (1), STARD9 (2), PALB2 (1), MYO19 (1)	AUTS2: Intellectual developmental disorder, autosomal dominant 26, OMIM # 615834, AD., {Autism susceptibility 15}, # 612100; CNTNAP5: Pitt-Hopkins like syndrome 1, OMIM # 610042, AR; DCDC2: Nephronophthisis 19, OMIM #616217, AR, Sclerosing cholangitis, neonatal, OMIM #617394, AR; GRIN2A: Epilepsy, focal, with speech disorder and with or without impaired intellectual development, # 245570, AD; GRIN2B: Developmental and epileptic encephalopathy 27, OMIM # 616139, AD, Intellectual developmental disorder, autosomal dominant 6, with or without seizures, OMM # 613970, AD; KIAA0319: {Dyslexia, susceptibility to, 2} OMIM 600202 AD; OXR1:Cerebellar hypoplasia/atrophy, epilepsy, and global developmental delay, OMIM # 213000, AR; SETBP1: Mental retardation, autosomal dominant 29, OMIM # 616078, AD, Schinzel-Giedion midface retraction syndrome, OMIM # 269150, AD; SRXP2:?Rolandic epilepsy, impaired intellectual development, and speech dyspraxia, X-linked, (RESDX) OMIM#30064;
Chui	2011	Novel interstitial duplication of 7p22.1 (46,XY.ish subtle(41 × 2). arr 7p22.1 (5,092,748–6,797,449) × 3 (*n* = 1); This region contains 27 genes, 13 of which are OMIM annotated (WIPI2, SLC29A4, FBXL18, ACTB, FSCN1, RNF216, OCM, PMS2, AIMP2, CYTH3, RAC1, KDELR2, ZNF12)	WIPI2: Intellectual developmental disorder with short stature and variable skeletal anomalies # OMIM 618453 AR; ACTB: Baraitser-Winter syndrome 1 OMIM 243310 AD; RNF216: Cerebellar ataxia and hypogonadotropic hypogonadism OMIM 212840 AR; PMS2: Mismatch repair cancer syndrome 4 OMIM 619101 AR; AIMP2: Leukodystrophy, hypomyelinating, 17 OMIM 618006 AR; RAC1:Mental retardation, autosomal dominant 48 OMIM 617751 AD; KDELR2: Osteogenesis imperfecta 21 619131 AR
IJlst	2002	AUH (patient 1: a homozygous IVS8-1G>A mutation, which disrupts the consensus sequence of the splice acceptor site; patient 2: apparently homozygous nonsense mutation, 589C>T. PCR-RFLP analysis on genomic DNA showed heterozygosity for the 589C>T mutation, indicating that the other allele is a null allele or produces an unstable mRNA	3-methylglutaconic aciduria, type I OMIM 250950 AR
Kalnak	2018	16p11.2 (*n* = 2, CNV loss); 17q12 (*n* = 1, CNV gain); 18p11.32-p11.22 (*n* = 1, CNV loss); 47,XXY (*n* = 1); Xp22.31-Xp22.33 (*n* = 1)	16p11.2: associated with a highly penetrant form of isolated severe early-onset obesity as well as obesity with developmental delay
Kwasnicka	2005	Inherited paracentric inversion of the long arm of chromosome 3 [46XX, inv(3)(q25.32–q29); novel cation transporter ATPase gene (ATP13A4) interrupted by 3q25–q29 inversion (*n* = 1)	
Lai	2000	patient 1 (same patient as Lai 2001): *de novo* balanced reciprocal translocation t(5;7)(q22;q31.2); patient 2: *de novo* balanced reciprocal translocation, t(2;7)(p23;q31.3)	Same patient described in Lai 2000
Lai	2001	SPCH1 (7q31; *n* = 1 (same patient described in Lai 2000)	Evidence that this form of speech and language abnormality (SPCH1) is caused by heterozygous mutation in the FOXP2 gene (605317) on chromosome 7q31. FOXP2: Speech-language disorder-1 602081 AD
LeGoff	2013	SRCAP (*n* = 9; 4 of these children have mild intellectual disability; 5 speech delay)	Developmental delay, hypotonia, musculoskeletal defects, and behavioral abnormalities OMIM 619595 AD 3; Floating-Harbor syndrome 136140 AD
Moralli	2015	Complex rearrangement involving an inversion of chromosome 7, followed by a translocation between the inverted chromosome 7 and chromosome 11 {(46, XX, der(7)inv(7)(p15;q31) t(7;11)(q21;p12), der(11)t(7;11)(q21;p12)}; The breakpoint on 7q31 mapped 200 kb downstream of FOXP2 (*n* = 1)	FOXP2 Speech-language disorder-1 OMIM 602081 AD - 7q31.1,?
Pettigrew	2015	De novo BP3-BP5 deletion at chromosome 15q13.1–13.3 (*n* = 1)	15q13.1; intellectual disability; 15q13.1: mild to moderate mental retardation or learning difficulties, or may have no cognitive deficits
Rakonjac	2015	22q11DS (*n* = 11)	
Riccardi	2015	Concurrent presence of an interstitial deletion in 13q34 (mat) and a terminal deletion in 4q35.2 (pat) (*n* = 2, siblings); The deleted region on chromosome 13 involves several genes (ATP11A, MCF2l, F7, F10, PROZ, PCID2, CUL4A, and LAMP1) The terminal deletion in 4q35.2 contains other OMIM genes (FRG1, FRG2 and DBET); dupl. Xp21.1 (mat); in 1 case additional pathogenic variant in PTPN11; parents not affected	F7: Factor VII deficiency OMIM 227500 AR; F10: Factor X deficiency OMIM 227600 AR
Thompson	1986	Pt 1: 46, XX, del(18)(qter p11?); pt 2: 45, XX, −13, −18, +t (13;18) (13 qter cen 18 qter); pt 3: 45, XX, −14, −18, +t(14;18) (14 qter cen 18 qter)	
Tomblin	2009	FOXP2 (*n* = 2, mother and daughter)	
Unger	2007	Sequencing of the FLNA gene revealed heterozygosity for a single missense mutation (c.3872C>T) in exon 23 which is predicted to cause an amino acid substitution in filamin repeat 11 (Pro1291Leu) (*n* = 1)	Xq28: FLNA: FG syndrome 2 OMIM 300321 XL 3 FLNA
Weihstuch	1996	46,XY, t{2q;lq}) (*n* = 1)	
Yeung	2013	DHP; two missense mutations were found: a novel c.48C>G transversion that changes asparagine to lysine at codon 15 in exon 1 (p.N16K), and a c.905G>A transversion that changes arginine to glutamine at codon 302 in exon 5 (p.R302Q) (*n* = 1)	DHP: Dihydropyrimidinuria OMIM 222748 AR

^a^ *n* can differ from total number of patients studied (e.g., in some patients no genetic diagnosis was made).

Reported SCAs related to DLD were 45,X (Turner-syndrome); 47,XXY (Klinefelter-syndrome), 47,XYY; 47,XXX; 48,XXYY; 48,XXXX; 48,XXXY; 49,XXXXX;49,XXXXY; XX/XO, XY/XXY mosaicism, and a ring Y-chromosome.

#### Chromosomal aberrations

Seven studies (3 case reports, 2 case series and 1 cohort study) reported on an identified chromosomal anomaly encompassing a chromosomal deletion (43), chromosomal translocations (*n* = 5) (44–46), chromosomal inversions (*n* = 2) (47, 48), and a complex chromosomal rearrangement (49), for details see Table 3B.

##### Chromosomal deletion 18p

Thompson and colleagues (43) discussed three children with a chromosome 18p deletion. All children presented with a speech and (both expressive and receptive) language delay with a striking delayed articulation. There was a marked difference between verbal and non-verbal abilities. The non-verbal IQ in one case was 90, compared to 60 and 68 in the two other cases. The clinical features were consistent with the 18p- syndrome, including short stature and facial dysmorphisms.

##### De novo chromosomal structural aberration

Bogart and colleagues (47) presented a girl with a prenatally detected *de novo* complex chromosomal rearrangement karyotype: 46,XX,t(6;11) (p21;q21),t(11;21) (q21;p13),inv(6) (p21q11). Her “speech development” was delayed. At 2.5 years of age, she spoke her first words. At 2 ¾ years, she did not make complete sentences. Growth was normal until 30 months of age. Eventually, growth was below the 5th centile. No further follow-up was presented.

Weistuch and colleagues (44) reported on a five-year-old boy with a balanced chromosomal translocation (46, XY, t(2q;1q) and a “specific expressive language impairment with verbal apraxia”. Other developmental milestones were normal. No specific physical features were mentioned.

##### Chromosomal rearrangement at 7q31

Lai and colleagues (45) discussed two cases with different chromosomal translocations, but with a similar chromosomal breakpoint (the SPCH1-region on 7q31) (45). This locus corresponded with the locus co-segregation in the affected family members in the large three-generation pedigree of the well-known K.E.-family, (K.E.), in which a severe speech and language disorder is transmitted as an autosomal dominant monogenic trait. In a second included publication of the same author, Lai and colleagues identified *FOXP2* as the causative gene for the language disorder in the KE-family and in one of the above included patients, unrelated to the KE-family (50).

Moralli and colleagues (49) reported on a young female, presenting with a severe speech and language disorder with a non-verbal IQ above the mean for her age, with a novo complex chromosomal rearrangement with breakpoint on 7q31, mapping 200 kb downstream of the gene *FOXP2*. Although no splice site or non-synonymous coding variants could be found in the *FOXP2* coding sequence, expression of some of the *FOXP2* targets (*EFNB2*, *INHBB*, *NTN4*, *ROBO2*, and *SLC14A1*) were altered, suggesting a possible role of *FOXP2* and/or its downstream targets in the etiology of the speech and language disorder in this girl.

Tomblin and colleagues (46) discussed a mother and daughter with DLD due to a chromosomal translocation disrupting the gene *FOXP2*. The language phenotype of mother and daughter was more or less comparable with KE-family, both also demonstrated significant impairments of speech.

##### Chromosome inversion

Kwasnicka and colleagues (48) described a girl with an expressive and receptive language delay and a paracentric inversion of the long arm of chromosome 3 [46XX, inv(3)(q25.32–q29), leading to the identification of the gene *ATP13A4 (*transporter ATPase gene) as a possible cause of language delay.

#### Copy number variations (CNVs)

Seven studies reported on a CNV detected by array CGH or SNP array in cases with isolated DLD (51–57), for details see Table 3.

In one of these studies, by Centanni et al. (53), the chromosome region 15q11.2 was found to be a susceptibility region for “specific language impairment” based on genome wide analysis for CNVs in eight children aged 4–17 years with DLD without any comorbid conditions.

Ceroni and colleagues (52) presented the identification of a homozygous microdeletion of exon 5 in the gene *ZNF277*, which falls within the *AUTS1* locus, in a girl with “specific language impairment”. They performed a genome-wide CNV screen in 512 individuals from families with SLI, who all formed part of the SLI Consortium. Screening of an additional 321 SLI families showed an increased allelic frequency of *ZNF277* microdeletions. However, due to the rarity of the microdeletion this difference did not reach significance.

Chui and colleagues (51) described a boy with a “deficit … best characterized as significant isolated speech delay” with a dup7p22.1.

Kalnak et al. (57) analyzed rare and *de novo* CNVs in 58 children with severe DLD and their siblings. Clinically significant CNVs or chromosomal anomalies were found in 4 of these children, of which 2 carried 16p11.2-deletions. One sibling who also carried a deletion at this locus did not have any neurodevelopmental problems reported. The other two clinically significant variants that were found in affected probands were a 17q12 duplication and 47, XXY respectively.

Pettigrew and colleagues (54) identified a deletion of 15q13.1–13.3, as a possible cause of isolated DLD, in a girl with clinical concerns regarding speech and language development. She spoke her first words at 21 months (>1 SD of the cohort mean age at first word) and presented at 3 years and 7 months with difficulty producing speech-sounds coupled with problems of expressive and receptive language development. Her IQ was above 70. The deletion in this girl was *de novo* and no family history of language impairment or dyslexia was reported. Both parents and her sibling scored above average on cognitive tests.

Rakonjac et al. (55) compared speech and language abilities of children with a 22q11.2 microdeletion to children with a phenotype resembling 22q11.2DS but without the microdeletion. Their results revealed that children from the group with the microdeletion had a lower level of speech and language abilities compared to the group without the microdeletion.

Riccardi and colleagues (56) reported on a patient and a sibling with DLD, both with the same constellation of familial CNVs (dupXp22.11; del4q35.2 and del13q34). These CNVs were respectively inherited from an unaffected mother and father. However, one of the affected siblings had an additional pathogenic variant in *PTPN11*, associated with Noonan syndrome.

#### Gene variants

In total, seven studies discussed a variety of candidate genes and gene-variants (50, 58–63), for details see Table 3.

Addis and colleagues (59) presented a three-generation family with language impairment with “auditory processing difficulties” as a core deficit. They compared the results of this family with the results of a control group. All affected family members reported a language development delay. In all tested affected family members, auditory discrimination deficits for tone duration were detected by psychoacoustic tests. Non-verbal IQ was normal. Linkage analysis mapped the disorder to the chromosomal region 12p13.31-q14.3 with a maximum LOD score of 2.2. The haplotype at this locus fully co-segregated following an autosomal dominant pattern. Further sequencing of six relevant candidate genes in this region (*CNTN1*, *FOXJ2*, *GRIN2B*, *NELL2*, *NAB2*, *SRGAP1*) revealed no causative pathogenic gene variants. To exclude possible underlying causative CNVs, genome wide CNV analysis was performed, yielding no novel inherited copy number variants.

Andres and colleagues (63) performed whole-exome sequencing (WES) in a single family (*n* = 11). They identified co-segregating rare variants in three genes: *BUD13*, *APLP2*, and *NDRG2*. To determine the significance of these genes in SLI, the authors Sanger sequenced all coding regions of each gene in unrelated individuals with SLI (*n* = 175). Variants in *BUD13* reached genome-wide significance (*p*-value < 0.01) upon comparison with similar variants in the 1,000 Genomes Project, providing gene level evidence that *BUD13* is involved in SLI. Additionally, five *BUD13* variants showed cohesive variant level evidence of likely pathogenicity.

Chen and colleagues (61) reported on the results of broad genetic analysis in 43 unrelated probands with severe “specific language impairment”. These probands were selected from the SLI consortium cohort, recruited from five centers across the UK. The group of probands had a mean verbal IQ of 84.2 (−1.1 SD), compared to a mean non-verbal IQ of 98.7 (−0.1 SD). Expressive language was more affected than receptive language. The mean scores were 65.9 (−2.3 SD below expected for chronological age) and 73.8 (−1.7 SD) for expressive and receptive language respectively. Genome wide exome sequencing was performed, followed by Sanger validations and segregation analyses. In several cases, analysis of a pre-defined set of 19 known candidates implicated in language related syndromes, could identify probable pathogenic gene variants in the genes *ERC1*, *GRIN2A* and *SRPX2.* Potential pathogenic variants, identified in the genes *SEMA6D*, *AUT2* and *ROBO1*, co-segregated with the language disorder in affected relatives of the respective probands. Furthermore, novel variants were identified in *GRIN2B* and *CNTNAP2*. Six rare SNVs were identified in the genes *ATP2C2*, *AUTS2*, *CNTNAP5*, *ROBO1* and *SRPX2*. Analysis beyond the known candidate genes revealed 7 rare or novel stop-gain variants in the genes *OR6P1*, *NUDT16l*, *SYNPR*, *OXR1*, *IDO2*, *MUC6* and *OR52B2*. Two of these variants, in the genes *OXR1* and *MUC6*, showed co-segregation with the disorder in affected family members. Compound heterozygous variants were identified in 11 genes. Four cases carried more than one rare gene variants probably reflecting “multiple hits”. One proband showed a rare coding variant in *AUTS2*, with in addition a stop-gain in *OR52B2*, and a rare variant the genes *OR52B2*, *KIAA0586* (OMIM*610178), and *STARD9*.

Unger and colleagues (58) reported on a boy, 18 months of age, with a mild delay in language acquisition and a normal psychomotor development and a possible causative variant in the gene *FLNA*.

#### Metabolic gene variants

Two studies reported on cases with DLD with gene variants causative for a metabolic disorder. Yeung and colleagues (60) presented a 2-year-old boy diagnosed with Dihydropyrimidinase deficiency (OMIM # 222748); compound heterozygous for pathogenic variants in the gene *DPYS*. The boy could not produce a single recognizable word, although his motor development was appropriate for his age. Neurological examination was normal. He had no dysmorphic features.

IJlst and colleagues (62) reported on two patients with “retardation in speech development” diagnosed with 3-methylglutaconic aciduria type I (OMIM # 250950). In one of them a motor development delay became evident in retrospect. In both cases, homozygosity for a pathogenic variant in the gene *AUH* confirmed the diagnosis.

### Additional search OMIM reported DLD genes

The 45 genes found within the retrieved studies were evaluated within the OMIM database. By this it was revealed that 22 of these 45 DLD (candidate) genes were identified as a cause for a genetic disorder (see Supplementary Table S4).

Two of these genes, *FOXP2* and *GRIN2A*, are OMIM morbid genes strongly related to speech and language development. *ATP2C*2 is identified as a susceptibility locus (OMIM #606711). To date, *FOXP2* is well-known as monogenic cause for the autosomal dominant disorder Speech-language disorder 1 (*SPCH1*; OMIM # 602081).

Twenty-two of the 45 genes are a known cause for intellectual disability, 17 for epilepsy or seizures, 18 for autism spectrum disorder, and 8 for dyslexia.

Four out of 45 described genes do not seem to be directly related to speech and language development; for more details see Supplementary Table S4.

## Discussion

With this systematic review of the literature, we aimed to gain more insight into the genes found in children with a language disorder without differentiating conditions (e.g., intellectual disability, epilepsy, autism and anatomical issues like clefts).

Our search resulted in 47 studies that met the inclusion criteria and that subsequently were critically appraised. A total of 34 studies had a low risk of bias, 9 studies a moderate risk of bias and 4 studies a high risk of bias. The included studies displayed a large spectrum of genetic causes of DLD.

### Chromosomal anomalies and CNV's

Interestingly, more than half of the papers (25/47) concerned findings of SCA, including 45, XO (Turner syndrome) and 47, XXY (Klinefelter syndrome). The large number of papers on SCA can be explained by the high frequency of SCA in the general population and seems in line with the increasing evidence SCA is often associated with mild clinical features (64).

Fifteen out of the 47 papers reported on chromosomal anomalies (8/47) and CNV's (7/47) as a possible cause of DLD. Chromosomal rearrangements at 7q31, eventually, led to identification of the DLD gene *FOXP2*. Furthermore, a chromosomal inversion and an intragenic CNV revealed *ATP13A4* and *ZNF277*, respectively, as candidate genes for DLD.

The reported CNV's display both deletions and duplications on different chromosomes. The CNV 15q13.1-q13.3 deletion reported by Pettigrew and colleagues (54) and the 16p11.2 deletion reported by Kalnak and colleagues (57), respectively, was also, recently, mentioned by Plug and colleagues (12) as causative CNV's in their assessment of the genetic work-up in 127 patients diagnosed with DLD.

Interestingly, the region 15q11.2 was previously reported by Centanni and colleagues (53) as a susceptibility locus, thus not as a causative CNV for isolated DLD. They describe four cases with a 15q11.2 duplication who all had one or more additional CNV's (13q21.1 dup and 12p13.33 del; 10q21.1del and 16p11.2del; 9p24.3 and 22q13.33 and 7q11.23 dup, respectively). Moreover, these CNV's are often associated with a broader clinical spectrum including apraxia, autism, epilepsy and/or intellectual disability (65, 66).

In addition, the 17q12 duplication and 22q11.2 deletion reported in relation to LD by Kalnak and colleagues (57) and Rakonjac and colleagues (55), respectively, are associated with a broad and variable clinical spectrum with a mild to severe development delay [Mitchel et al. (67)]. In our review, studies on 22q11.2DS were generally excluded because of these and other risk factors.

The role of the reported combination of familial CNVs (dupXp22.11; del4q35.2 and del13q34) in the cause of DLD (56) can be argued, because one of the sibs of the proband was described to be diagnosed with Noonan syndrome. On the other hand, one might hypothesize these CNV's contribute to a multifactorial etiologic model.

### (Candidate) genes

From the 21 selected papers not related to SCAs, 45 (candidate) genes for DLD, including *FOXP2*, *ATP2C2, ERC1*, *GRIN2A* and *KMT2D* (Tables 3B, Supplementary Table S4).

Most of these genes (31 out of 45) were reported and discussed by Chen and colleagues (61).

Of these, *FOXP2* is acknowledged as a monogenic cause for specific Speech-Language disorder-1 (OMIM# 602081) and *ATP2C2* as a susceptibility locus (OMIM % 606711) contributing in the etiology of DLD. On the other hand, *ERC1* was supported by the authors as an interesting candidate for DLD, by the identification of a novel probably pathogenic variant in a child with DLD and its affected mother. However, since the father also reported a history of DLD and both sibs needed special education, although they were not carrying the *ERC1* variant, the exact inheritance pattern still needs to be unraveled. *KMTD2* was also identified as candidate gene. The authors hypothesized specific missense variants might lead to DLD, because the described cases did not show features of Kabuki syndrome (OMIM#147920), caused by loss of function in *KMTD2* gene.

The genes *SEMA6D*, *AUT2* and *ROBO1*, *OXR1* and *MUC6* were reported as interesting candidate genes because potential pathogenic variants co-segregated with the language disorder in affected relatives of the respective probands (61). The genes *FAT3*, *KMTD2*, *SCN9A* and *PALB2* were considered as interesting candidate genes because compound heterozygous potential deleterious variants, inherited from the opposite parent, were identified in these genes (61). Most of the reported genes, when checked in OMIM, were also related to intellectual disability, epilepsy and/or autism. This might be related to the study design. Chen and colleagues first focused on a pre-defined set of known candidates from the literature, and identified potentially pathogenic variants in genes already implicated in diverse language-related syndromes, including *ERC1*, *GRIN2A*, and *SRPX2*. They performed whole-exome sequencing in 43 unrelated probands affected by severe specific language impairment, followed by independent validations with Sanger sequencing, focusing on these candidate genes.

Andres and colleagues (63) found gene level evidence that *BUD13* is involved in SLI.

Further studies are needed to support the exact contribution of the reported candidate genes in the etiology of isolated DLD nd to gain more insight in to what extent these genes can be defined as a monogenic cause or a contributing factor in a multifactorial etiology of DLD. The identification of more than one rare gene variant probably representing “multiple hits” in four cases, support a multifactorial inheritance in some cases of DLD (61).

### Clinical spectrum

Notably, 22 out of the 45 reported (candidate) genes are known to be related to a broader phenotype than DLD, including intellectual disability, epilepsy, and/or autism (e.g., *GRIN2A*, *CNTNAP2*, *CNTNAP5*, *AUTS2*). Even *FOXP2*, often considered the “language gene” and in OMIM recognized as specific Speech-Language disorder-1 (OMIM; # 602081), has been linked to other neurodevelopmental disorders, like intellectual disability and autism (68, 69).

Although most of the study designs used in the included papers were case-related, and symptoms in individual cases might emerge at a later stage, the results in this review suggest that the etiology of DLD may be heterogeneous, and that DLD can actually be part of a broader phenotype.

Interestingly, this systematic review revealed two papers reporting cases with apparently DLD and pathogenic gene variants in the genes *DPYS* and *AUH*, which are known to be responsible for the metabolic disorders Dihydropyrimidinase deficiency (OMIM # 222748) and 3-methylglutaconic aciduria type I (OMIM # 250950), respectively (60, 62). These cases might demonstrate that both metabolic disorders can result in a mild to severe phenotype. However, the absence of significant clinical features in the reported cases, besides DLD, might be related to their young age.

In conclusion, this systematic review supports the theory that DLD is a heterogeneous disorder and that different genetic underlying mechanisms, including SCA, CNV's, and a variety of gene variants, can play a role in the etiology of DLD. Common underlying etiologic mechanisms seem to be involved in a broader phenotypic spectrum which includes DLD. If this is the case, DLD could be the first manifestation of a broader underlying etiology, and this would mean that it could constitute a starting point for further genetic diagnostics to unravel the full spectrum of the disorder.

### Genetic diagnostics

As a result of the increasing awareness of a possible genetic cause for DLD and the technological advancements made in genetic analysis, DNA analysis is increasingly being implemented in the diagnostic work-up in patients with DLD seen in clinical practice.

An early genetic diagnosis can be important for early treatment and better outcomes. For example, DLD might be a first clinical presentation of epileptic activity without clinically apparent seizures (70), which needs treatment to improve outcomes. Also, other genetic disorders, like SCAs (e.g., 47, XXY, Klinefelter syndrome and 45, XO; Turner syndrome) and microdeletions (e.g., 22q11.2 deletion), require a specific management and adequate follow-up. For example, early hormonal therapy was found to be associated with a positive effect on, amongst others, expressive and receptive language in boys with Klinefelter syndrome (71).

When genetic testing in children with DLD is considered, we recommend to start with SNP array analysis based on the frequently reported chromosomal disorders as the cause of DLD. Nowadays, SNP array, identifying sex chromosomal disorders and microdeletions/-deletions, is broadly implemented in clinical care. Next generation sequencing (NGS) can detect underlying pathogenic gene variants. It has been demonstrated that NGS, analyzing a large number of genes simultaneously, proves to be highly efficient and cost-effective (72, 73). By implementation of novel analysis tools, CNVs can also be detected by NGS.

Especially in patients with DLD with a significant discrepancy in IQ with the parents, with a high IQ in the parents, broader genetic testing by NGS should be considered. In these cases, a *de novo* CNV or *de novo* pathogenic variant in a gene associated with intellectual disability might be present.

### Strengths and limitations

Although we thoroughly evaluated the literature on developmental language disorder to create unique knowledge on genetic causes of DLD, we realize this study has some important limitations. An important issue is the fact that different definitions of DLD are used in different studies. Furthermore, as the main topic of the study in most cases was not language development, it is not always clear how language was measured and how isolated the language problems really were. Based on the outcomes and descriptions of cases in the included studies, it was not possible to reach a good overview of possible differences in language phenotype between the different underlying genetic causes, which could be used in a more personalized genetic diagnostic work-up in clinical practice.

In addition, since techniques for clinical genetic diagnostics have evolved rapidly during the last years, aberrations that can be detected by relatively “old” techniques like karyotyping (e.g., SCAs) may be overrepresented in the search query, simply as a consequence of a greater number of studies performed using this technique because of the larger time frame. Aneuploidies can be detected since the 1950s by general karyotyping (74) and array CGH detecting CNV's is developed in 1992. The earliest study included in this review was the study of Garvey, from 1973 (25).

Finally, only articles with full texts in Dutch and English were included, as these are the languages all reviewers have a good command of, which means that we might have missed an important article in any other language.

### Future research

With these limitations in mind, our review clearly exemplifies that DLD that appears to be isolated can have an underlying genetic etiology and that genetic testing should be considered in the diagnostic work-up in patients with DLD without any apparent comorbidities. More knowledge on possibly different developmental language profiles associated with different genetic genotypes could be useful in adequate and efficient genetic counseling.

It would be helpful if in studies regarding a genetic disorder which is associated with DLD, a consistent terminology is used to describe the language profile of the patient and more details on the parents are given. A more elaborate description of the language profile associated with specific genotypes in combination with the relevant gene function could also lead to a better understanding of the neurobiological causes of DLD.

Larger and more controlled studies like cohort studies directed at specific genetic variants and their relation to a deviant language development in children are recommended.

## Conclusion

In this systematic review, we found 47 studies reporting on genetic findings found in children with isolated DLD. Twenty-five were related to numerical sex chromosome aberrations. The other 22 papers concerned a highly diversified group of CNV's and (candidate) genes. A possible explanation for the low yield would be that if a genetic background for DLD exists, the phenotype is broader and also includes (potential partially explanatory) issues like intellectual disability, epilepsy, and/or autism. Besides this, at the current stage, genetic diagnostic testing is not part of standard diagnostic work-up in children with DLD. Based on the findings of our review, we hypothesize that in most cases DLD has a multifactorial inheritance and not a monogenic etiology. DLD and LD associated with comorbidities could have an overlapping genetic etiology, in which case DLD could be the first manifestation of a broader underlying etiology.

When genetic testing in children with DLD is considered, we recommend to start with SNP array analysis based on the frequency of chromosomal disorders as the cause of DLD found in this study.

Especially in patients with DLD with a significant discrepancy in IQ with the parents, with a high IQ in the parents, broader genetic testing by NGS should be considered.

More research is needed regarding the questions what role clinical genetic research could play in diagnosing DLD, the relation between genetic cause and DLD profile or treatment outcome, and the preferences or perspectives of patients/parents on genetic testing for DLD.

## Data Availability

The original contributions presented in the study are included in the article/Supplementary Material, further inquiries can be directed to the corresponding author.

## Appendix 1

Search queries used are:

PubMed

(((((genetics[MeSH Terms]) AND syndrom*[Title/Abstract])) OR (((((((gene[Title/Abstract]) OR genes[Title/Abstract]) OR genetic*[Title/Abstract]) OR chromosom*[Title/Abstract]) OR mutation*[Title/Abstract]) OR human genetics[MeSH Terms])))) AND (((((((((((delay*[Title/Abstract]) OR impair*[Title/Abstract]) OR disorder*[Title/Abstract]) OR abilit*[Title/Abstract]) OR problem*[Title/Abstract]) OR development*[Title/Abstract])) AND language[Title/Abstract])) OR ((((((((delay*[Title/Abstract]) OR impair*[Title/Abstract]) OR disorder*[Title/Abstract]) OR abilit*[Title/Abstract]) OR problem*[Title/Abstract]) OR development*[Title/Abstract])) AND speech[Title/Abstract])) OR language disorder[MeSH Terms])

Embase

#1: Language

("Language Disorders"[Mesh] OR language impairment[Title/Abstract] OR communication disorder[Title/Abstract] OR semantic disorder[Title/Abstract] OR syntactic disorder[Title/Abstract] OR speech disorder[Title/Abstract] OR sound disorder[Title/Abstract] OR phonological disorder[Title/Abstract] OR speech impairment[Title/Abstract] OR speech delay[Title/Abstract] OR language delay[Title/Abstract] OR pragmatic disorder[Title/Abstract] OR language problem[Title/Abstract] OR language difficulties[Title/Abstract] OR language difficulty[Title/Abstract] OR language retardation[Title/Abstract] OR communication disorder[Title/Abstract] OR communication delay[Title/Abstract] OR language disorder[Title/Abstract] OR language learning[Title/Abstract] OR language development[Title/Abstract] OR language abilities[Title/Abstract] OR language ability[Title/Abstract])

AND

#2: Genetics

(genes[Title/Abstract] OR genetic[Title/Abstract] OR chromosome[Title/Abstract] OR chromosomes[Title/Abstract] OR chromosomal[Title/Abstract] OR syndrome[Title/Abstract] OR syndromes[Title/Abstract] or syndromal[Title/Abstract] OR mutation[Title/Abstract] OR heredit*[Title/Abstract] OR inherit*[Title/Abstract] OR "Genetic Diseases, Inborn"[Mesh] OR "Genes"[Mesh] OR "Genetic Loci"[Mesh] OR "Inheritance Patterns"[Mesh])

AND

#3: Children

("Infant"[Mesh:noexp] OR "Child"[Mesh] OR infant[Title/Abstract] OR infants[Title/Abstract] OR toddler[Title/Abstract] OR child[Title/Abstract] OR children[Title/Abstract] OR boy[Title/Abstract] OR girl[Title/Abstract])
