# Supplementary material for: Genetic outcomes in children with developmental language disorder: a systematic review
Source: Front Pediatr. 2024 Jan 17;12:1315229. doi: 10.3389/fped.2024.1315229 (PMC10828955; doi:10.3389/fped.2024.1315229)
Supplement: Supplementary file 1 [file Table4.docx]

| **Gene**  Chromosome locus  (study) | **OMIM gene** | **OMIM**  **Phenotype/disorder** | **DLD gene** | **ID gene** | **Autism gene** | **Epilepsy gene** | **Dyslexia** | **other** |
| --- | --- | --- | --- | --- | --- | --- | --- | --- |
| *FOXP2*  7q31.1  (Lai et al, 2001; AD family  Tomblin et al, 2009; 2 families) | * 605317 | Speech-language disorder-1  OMIM # 602081 | +  OMIM # 602081 | +  (OMIM # 602081)  (Reuter et al 2017  PMID: 27572252)  Mild cognitive impairment was reported for most individuals.  Morgan et al 2017  PMID: 27336128 | +  Chen et al 2020  PMID: 32912122  Morgan et al 2017  PMID: 27336128 | +  Morgan et al 2017  PMID: 27336128 | + ^a^  Doust et al 2020  PMID: 32248883 | - |
| ***GRIN2A***  16p13.2  (Chen et al,  2017;  n=1; novel variant, Co-segr) | * 138253 | Epilepsy, focal, with speech disorder and with or without impaired intellectual development, # 245570, AD | +  OMIM # 245570  Strehlow et al 2019  PMID: 30544257 | +  OMIM # 245570  Strehlow et al 2019 PMID:30544257 | +  OMIM # 245570  Strehlow et al 2019 PMID:30544257 | +  OMIM # 245570 | - | Verbal dyspraxia  Strehlow et al 2019 PMID:30544257 |
| ***ATP2C2***  Chr16q24.1  (Chen et al,  2017, total n=6;  n=2 pop. freq. <1%; Co-segr, n=2pop.freq. 1-5%, n=2 pop.freq >5%) | * 613082 | Specific language impairment QTL, 1, OMIM % 606711 | + ^a, gv^  OMIM % 606711  Martinelli et al 2021  PMID: 33864365  Smith et al 2015 PMID: 25296922  Chen et al,  2017 | - | - | - | + ^a^  PMID: 29201552  Muller et al 2017;  ATP2C2 and DYX1C1 are putative modulators of dyslexia-related MMR | - |
| **Candidate Gene** |  |  |  |  |  |  |  |  |
| *ATP13A4*  3q29  (Kwasnicka-Crawford et al, 2005;  n=1 and cohort`) | * 609556 |  | + ^c^  Kwasnicka-Crawford et al, 2005 | - | + ^cnv^  Lesca et al 2012  PMID: 22738016  Kwasnicka-Crawford et al, 2005 | + ^cnv^  Lesca et al 2012  PMID: 22738016  Kwasnicka-Crawford et al, 2005 | - | Verbral apraxia gv  Worthy et al 2013  PMID: 24083349 |
| *AUTS2*  7q11.22  (Chen et al,  2017, totl n=2; n=1, variant pop.freq <1% in addition to stop-gain in OR52B2, and  multiple rare variants in each of the OR52B2, KIAA0586, STARD9, n=1 variant pop.freq >5%, co-segr) | * 607270 | Intellectual developmental disorder, autosomal dominant 26, OMIM # 615834, AD | + ^gv *^  Chen et al,  2017 | +  OMIM# 615834 | + ^f^  Hori et al 2017  PMID: 28505103 | - | - |  |
| *BIRC6*  2p22.3  (Chen et al,  2017,  n=2, compound heterozygous, and rare variant in addition to other variants) | * 605638 |  | + ^gv *^  Chen et al,  2017 | - | + ^chr^  Costa et al 2022  PMID: 34664255 | - | - | - |
| *BUD13*  Chr11:116633616  (Andres et al. 2021; n=4 from 1 family) |  |  |  | +Karnak er al. 2022 |  |  |  |  |
| *CNTN1*  12q12  (Addis et al, 2010, n=3 from 1 family, variant) | * 600016 | ?Myopathy, congenital, Compton-North, OMIM 612540, AR | + ^gv? **^  ^Addis et al, 2010^ | - | - | - | - | - |
| *CNTNAP2*  7q35-q36.1  (Chen et al,  2017,  n=1, novel variant) | * 604569 | Pitt-Hopkins like syndrome 1, OMIM # 610042, AR  {Autism susceptibility 15}, # 612100 | + ^gv *^  Chen et al,  2017 | +  OMIM# 610042 | + ^chr, cnv, gv^  OMIM# 612100 | +  OMIM# 610042 | +  Gu et al 2018  PMID: 30017804 | Verbral apraxia ^gv^  Worthy et al 2013  PMID: 24083349 |
| ***CNTNAP5***  2q14.3    (Chen et al,  2017, total n=2: n=1, variant pop.freq 1-5%, n=1, variant pop.freq <1%) | * 610519 |  | + ^chr, cnv, gv *^  Balaratti et al 2009  PMID: 19236961  Chen et al 2017 | + ^chr, cnv^  Balaratti et al 2009  PMID: 19236961  Aleo et al 2020  PMID: 32975021 | + ^chr, cnv, gv^  Aleo et al 2020  PMID: 32975021  Pagnamenta et al 2015  PMID: 20346443 | + ^chr, cnv^  Balaratti et al 2009  PMID: 19236961 | - | - |
| *DCDC2*  6p22.3  (Chen et al,  2017, total n=3, n=1, variant pop.freq >5%, n=2 variant pop.freq 1-5%) | * 605755 | Nephronophthisis 19, OMIM #616217, AR,  Sclerosing cholangitis, neonatal, OMIM #617394, AR, | + ^cnv^  Eicher 2016  PMID: 25953057 | +^gv^  Syryn 2021  PMID: 34155636 | - | - | + ^a^  Marino 2012  PMID:21881542  Eicher 2016  PMID: 25953057 | Two cases of DCDC2-related neonatal sclerosing cholangitis with developmental delay  Syryn 2021  PMID: 34155636 |
| *EFNB2*  13q33.3  (Moralli et al 2015;  n=1, complex chromosomal rearrrangement with breakpoint on 7q31 mapped 200 kb downstream of FOXP2, altered gene expression in presence of EFNB2) | * 600527 |  | + ^chr^  Moralli et al, 2015 | +^cnv, gv^  (Lévy et al 2018; PMID: 29508392) | - | + ^cnv, gv^  (Lévy et al 2018; PMID: 29508392) |  | - |
| ***ERC1***  12p13.33  (Chen et al,  2017; total  n=3, n=1 pop.freq. >5%, n=1 pop.freq 1-5%, n=1, n=1 novel start-loss) | * 607127 |  | + ^gv *^  Chen et al,  2017 | + ^cnv,^  Thevenon et al 2013  PMID: 22713806 | - | - | - | Verbal apraxia ^cnv,^  Thevenon et al 2013  PMID: 22713806 |
| *FAT3*  11q14.3    (Chen et al, 2017;  n=1, compound heterozygous variants, co.segr.) | * 612483 |  | + ^gv *^  Chen et al,  2017 | + ^gv^  Baldwin et al 2021  PMID: 33562221  (candidate gene) | + ^gv^  Baldwin et al2021  PMID: 33562221  Rochtus et al2020  PMID: 31957018  (candidate gene) | + ^gv^  Rochtus et al2020  PMID: 31957018  (candidate gene) | - | - |
| *FLNB*  3p14.3    (Chen et al, 2017;  n=2, 2 compound heterozygous rare variants) | * 603381 | Atelosteogenesis, type I, OMIM 108720, AD; Atelosteogenesis, type III, OMIM # 108721, AD;  Boomerang dysplasia, OMIM # 112310, AD;  Larsen syndrome, OMIM # 150250, AD;  Spondylocarpotarsal synostosis syndrome, OMIM # 272460, AR | - ^gv *^  Chen et al,  2017 | - | - | - | - |  |
| ***GRIN2B***  12p13.1  (Chen et al,  2017,  n=1, novel variant) | * 138252 | Developmental and epileptic encephalopathy 27, OMIM # 616139, AD;  Intellectual developmental disorder, autosomal dominant 6, with or without seizures, OMM # 613970, AD | + ^gv *^  Chen et al,  2017 | +  OMIM # 616139  OMIM # 613970 | +  OMIM # 616139  OMIM # 613970 | +  OMIM # 616139  OMIM # 613970 | + ^a^  Liu et al 2020  PMID: 32937172 |  |
| *IDO2*  8p11.21  Chen et al,  2017,  n=1, stop gain variant, functional signicant?) | * 612129 |  | - ^gv *^  Chen et al,  2017 | - | - | - | - | - |
| *INHBB*  2q14.2  (Moralli et al 2015; n=1, complex chromosomal rearrangement with breakpoint on 7q31 mapped 200 kb downstream of FOXP2, altered gene expression INHBB) | * 147390 |  | - ^chr^  ^Moralli et al, 2015^ | - | - | - |  | - |
| *KIAA0319*  6p22.3  (Chen et al,  2017; total n=6; n=2, variant pop.freq.1-5%, n=4, variant pop.freq. >5%)  (Andres et al, 2021) | * 609269 |  | + ^gv *^  Chen et al,  2017 | - | - | - | + ^a^  Deng et al 2019  PMID: 31204720 | Verbral apraxia ^gv^  Worthy et al 2013  PMID: 24083349 |
| *KIAA0586*  14q23.1  (Chen et al,  2017;  n=1, compound heterozygous variants- in addition to variant in AUTS2, stop-gain in OR52B2, and multiple rare variants in each of the OR52B2, and STARD9) | * 610178 | Joubert syndrome 23, OMIM # 616490, AR;  Short-rib thoracic dysplasia 14 with polydactyly, OMIM # 616546, AR, | + ^gv *^  Chen et al,  2017 | +  OMIM # 616490 | - | +  OMIM # 616490 | - |  |
| *KMT2D*  12q13.12    (Chen et al,  2017,  n=1, compound heterozygous variants, in addition to variant in STARD9 OMIM * 614642) | * 602113 | Kabuki syndrome 1, OMIM # 147920, AD | + ^gv *^  Chen et al,  2017 | +  OMIM # 147920 | +  OMIM # 147920 | +  OMIM # 147920 | - |  |
| ***MUC6*** ^#^  11p15.5  Chen et al,  2017,  n=1, stop gain variant, co. segr.) | * 158374 |  | + ^gv *^  Chen et al,  2017 | - | - | - | - ^a^  Hsiung et al 2004  PMID: 14755455 | - |
| *MYO16*  13q33.3  (Chen et al,  2017,  n=1, compound heterozygous variants) | * 615479 |  | + ^gv *^  Chen et al,  2017 | + ^cnv^  Liu et al. 2016  PMID: 25902260 | + ^cnv^  Liu et al. 2016  PMID: 25902260  Bugyi and Kengyel  2020  PMID: 32451869 | - | - | - |
| *MYO19*  17q12  (Chen et al,  2017;  n=1, compound heterozygous variants) | * 617379 |  | + ^gv *^  Chen et al,  2017 | - | - | - | - | - |
| *NFXL1*  4p12  (Chen et al,  2017;  n=1, variant pop.freq.>5%) | - |  | + ^gv *^  Chen et al,  2017  Nudel et al 2016  PMID: 27053962  Villanueva et al  2015  PMID: 25781923 | - | - | - | - | - |
| *NTN4*  12q22  (Moralli et al 2015; n=1, complex chromosomal rearrangement with breakpoint on 7q31 mapped 200 kb downstream of FOXP2, altered gene expression NTN4) | * 610401 |  | -  Moralli et al 201 | - | - | - | - | - |
| ***NUDT16L1***  16p13.3  (Chen et al,  2017,  n=1)) | * 617338 |  | + ^gv *^  Chen et al,  2017 | - | - | - | - | - |
| ***OR52B2*** #  11p15.4  (Chen et al,  2017;  n=1, co-segr. stop gain variant, in addition to variants in AUTS2, OR52B2, KIAA0586 (OMIM*610178) and STARD9) | - |  | + ^gv *^  Chen et al,  2017 | - | - | - | - | - |
| *OR6P1* #  1q23.1  (Chen et al,  2017;  n=1; stop gain variant, functional signicant?) | - |  | - ^gv, f?^  Chen et al,  201 | - | - | - | - | - |
| ***OXR1***  8q23.1  (Chen et al,  2017, n=1; stop gain variant, co-segr.) | * 605609 | Cerebellar hypoplasia/atrophy, epilepsy, and global developmental delay, OMIM # 213000, AR | + ^gv *^  Chen et al,  2017 | +  OMIM #213000  Wang et al 2019  PMID: 31785787 | - | +  OMIM #213000  Wang et al 2019  PMID: 31785787 | - |  |
| *PALB2*  16p12.2  (Chen et al,  2017; n=1, compound heterozygous variants) | * 610355 | Fanconi anemia, complementation group N, OMIM# 610832, AR  [Fanconi Anemia - GeneReviews® - NCBI Bookshelf (nih.gov)/](https://www.ncbi.nlm.nih.gov/books/NBK1401/) Developmental delay and/or intellectual disability is seen in 10%. | + ^gv *^  Chen et al,  2017 | +  OMIM # 610832 | - | - | - |  |
| ***ROBO1***  3p12.3  (Chen et al,  2017; total n=2; n=1, variant pop. freq. <1%; n=1, variant pop.freq. 1-5%; Co-segr.) | * 602430 |  | + ^gv *^  Chen et al,  2017 | - | - | - | + ^f^  Sun et al 2017  PMID: 28240421 | -  Devasenapathy et al 2018  PMID: 30199849  +  Bates et al 2011  PMID: 20949370 |
| *ROBO2*  3p12.3  (Moralli et al 2015; n=1, breakpoint on 7q31 mapped 200 kb downstream of FOXP2, altered gene expression ROBO2 | * 602431 | Vesicoureteral reflux 2, OMIM  # 610878, AD | + ^a^  SNP near ROBO2  (St Pourcain ea. 2014 PMID: 25226531) | - | - | - |  |  |
| *SCN9A*  2q24.3  (Chen et al,  2017;  n=1, compound heterozygous) | * 603415 | Erythermalgia, primary, OMIM # 133020, AD,  Insensitivity to pain, congenital, OMIM # 243000, AR,  Neuropathy, hereditary sensory and autonomic, type IID, OMIM # 243000, AR,  Paroxysmal extreme pain disorder, OMIM # 167400, AD,  Small fiber neuropathy, OMIM # 133020, AD | + ^gv *^  Chen et al,  2017 | - | - | +  Zhang et al  2020  PMID: 31372899 |  |  |
| ***SEMA6D*** 15q21.1    (Chen et al,  2017, total n=3;  n=2, variant pop.freq. >5%, n=1 novel variant, in addition to rare stop-gain in the SYNPR; co-segr) | * 609295 |  | + ^chr,gv*^  Chen et al,  2017  Ercan-Sencicek et al 2012  PMID: 22266071 | - | - | - | - | - |
| *SETBP1* 18q12.3  (Chen et al, 2017,  n=5, pop.freq.>5%) | * 611060 | Mental retardation, autosomal dominant 29, OMIM # 616078, AD;  Schinzel-Giedion midface retraction syndrome, OMIM # 269150, AD | +  OMIM# 616078  Marseglia et al 2012  PMID: 22333924  Chen et al, 2017  Morgan et al. 2021  PMID: 33907317 | +  OMIM# 616078  Leonardi et al 2020  PMID: 33391157 | +  Leonardi et al 2020  PMID: 33391157 | +  Leonardi et al 2020  PMID: 33391157 | + ^a^  Perdue et al  2019  PMID: 30009840 | - |
| *SLC14A1*  18q12.3    (Moralli et al 2015; n=1, breakpoint on 7q31 mapped 200 kb downstream of FOXP2, altered gene expression SLC14A1 | * 613868 | Kidd blood group system  OMIM # 111000 | -  (Moralli et al 2015; | - | - | - | - |  |
| *SRCAP*  16p11.2  (LeGoff et al 2012; n=3 variant) | * 611421 | Developmental delay, hypotonia, musculoskeletal defects, and behavioral abnormalities, 619595, AD, 3  Floating-Harbor syndrome, 136140, AD, | + ^gv^  LeGoff et al 2012 | + ^gv^  Rots et al 2021  PMID: 33909990 | + ^gv^  Rots et al 2021  PMID: 33909990 | + ^gv^  Rots et al 2021  PMID: 33909990 | - | - |
| *SRGAP1*  12q14.2  (Addis et al. 2010, n=2 from 1 family (n=3), variant) | * 606523 | - | -^**^  Addis et al. 2010 | - | (+)  Pérez et al 2016  PMID: 27089953 | - | - | {Thyroid cancer, nonmedullary, 2}, OMIM 188470, AD, SMu |
| ***SRPX2***  Xq22.1    (Chen et al,  2017,  n=1, variant pop.freq. <1%.) | * 300642 | ?Rolandic epilepsy, impaired intellectual development, and speech dyspraxia, X-linked, (RESDX)  OMIM#300643, | + ^gv *^  Chen et al,  2017 | OMIM #300643 | OMIM #300643 | - | - | - |
| *STARD9*  15q15.2  (Chen et al,  2017; total  n=2, n=1 compundheterozygous in addition to rare coding variant in AUTS2, stop-gain in OR52B2, and  multiple rare variants in each of the OR52B2, KIAA0586; n=1 compound heterozygous, in addition to variant I KMT2D) | * 614642 |  | + ^gv *^  Chen et al,  2017 | + ^gv^  Okamoto et al  2017  PMID: 28777490 | - | + ^gv^  Okamoto et al  2017  PMID: 28777490 | - | - |
| *SYNPR*  3p14.2  (Chen et al,  2017, n=1; stop gain variant, functional signicant?) | - |  | + ^chr, gv*^  De la Hoz et al 2015  PMID: 26075115  Chen et al,  2017 | + ^chr^  De la Hoz et al 2015  PMID: 26075115 | + ^chr^  De la Hoz et al 2015  PMID: 26075115 | - | - | - |
| *ZNF277*  7q31.1  (Ceroni et al, 2014;  n=1, deletion exon 5) | * 605465 |  | + ^cnv intragene^  SLI locus/ 16q/  OMIM % 606711  (Ceroni et al, 2014  PMID: 25537359) | - | + ^cnv^  (Ceroni et al, 2014  PMID: 25537359) | - | - | - |
| **Metabolic disorder** |  |  |  |  |  |  |  |  |
| *AUH*  9q22.31  (n=2)  Ijlst et al,  2017 | * 600529 | 3-methylglutaconic aciduria, type I, OMIM # 250950, AR | +  Ijlst et al,  2017  Illsinger et al 2004  PMID: 15033206 | +  OMIM # 250950 | +  OMIM # 250950 | +  OMIM # 250950  Illsinger et al 2004  PMID: 15033206 | - | - |
| *DPYSD*  8q22.3  (n=1)  Yeung et al,  2013 | * 613326 | Dihydropyrimidinuria, OMIM # 222748, AR | +  OMIM # 222748 | +  OMIM # 222748 | +  OMIM # 222748  İnci et al 2021  PMID: 33605552 | +  OMIM # 222748 | - | - |

a: association study

chr: chromosomal anomaly case

Co-segr: co-segregated with disorder in affected relatives of the respective probands

CNV: CNV anomaly

f: functional studies

gv: gene variant

pop. Freq.: population frequency

?: Functional impact variant not reported

*= The group of probands had mean scores of 65.9 (− 2.3 SD below expected for chronological age) and 73.8 (− 1.7 SD) for expressive and receptive language respectively, and a mean verbal IQ of 84.2 (− 1.1 SD), compared to a mean non-verbal IQ of 98.7 (− 0.1 SD) in

line with the mean of the general population (all scores normalized to a population mean of 100 and SD of 15). No individual data on IQ presented (Chen et al et al. 2017)

^** : Only one change (CNTN1 exon 12 c.1529hetC→T) was found in all three affected individuals sequenced (Addis et al. 2016)^

Genes highlighted in bold represent genes with changes that are predicted to be functionally significant by Chen et al 2017

Genes highlighted in bold & underscore represent events of putative significance by Chen et al. 2017

# “ Olfactory receptor and mucin family genes are especially susceptible to false positive findings in next-generation sequencing, due to mapping artefacts (http://massgenomics.org/2013/06/ngs-false-positives.html). Although these variants were validated by Sanger sequencing, they should be interpreted with caution, as reported by Chen et al 2017
