# Supplementary material for: Genetic outcomes in children with developmental language disorder: a systematic review
Source: Front Pediatr. 2024 Jan 17;12:1315229. doi: 10.3389/fped.2024.1315229 (PMC10828955; doi:10.3389/fped.2024.1315229)
Supplement: Supplementary file 2 [file Table5.docx]

| **Study** | **Design** | **Aim** | **Patients** | **Isolated/non-isolated** | **Diagnosis confirmed by genetic testing?** | **Language test?** |
| --- | --- | --- | --- | --- | --- | --- |
| ***SCA*** |  |  |  |  |  |  |
| Garvey et al, 1973 | Case series  N=9 | Speech evaluation; effect chromosomal anomaly on speech | Patients with sex chromosomal anomalies | Isolated,  however some cases with minor decrease in IQ | yes | yes |
| Tennes et al  1977 | Case series  N=17 | Assessment early development | 47,XXY (12), 47,XYY (4), 46,XY/47,XXY mosaicism (1) | Isolated  On infant scales measuring mental development, the infants’ scores with one exception were in the average range at 5 to 6 months (Table III). At 9 and 18 months all scores were within normal limits but covered | yes | yes |
| Haka-Ikse et al,  1978 | Case series  N=42 | Evaluation early development | Patients with sex chromosomal anomalies | Isolated and non-isolated  No IQ determined, DQ as measure for development,  as mean of the population | yes | yes |
| Bender et al, 1983 | Case control  N= 41 propositi; 31 controls (siblings) | Speech evaluation | Patients with sex chromosomal anomalies | Unclear | yes | yes |
| Rovet et al, 1996 | Systematic review;  27 studies – 10 cohorts + own cohort study  N= 36 (29 completed the study) | Evaluation psychoeducational profile of boys with Klinefelter Syndrome | 47,XXY | Isolated  Mean PIQs ranged from 93-102 for KS and from 99-111 for controls. | yes | yes |
| Stemkens et al,  2006 | Case series  N=61 | Influence of X-chromosomal imprinting on the phenotype in Klinefelter syndrome?  Determined parental origin of the extra X by CAG repeat length of the androgen receptor | 47,XXY | Isolated and non-isolated  Lower IQ included  TIQ 89.0 (15.5) n= 54 VIQ 87.0 (16.1) n= 50 PIQ 92.4 (15.8) n=50 | yes | Yes, by a questionnaire with 2 items on speech development: - speech-/language skills as compared to children of the same age - need for special education or for supportive education |
| Weimer et al,  2006 | Case report  N=1 | Evaluation phenotype | In Klinefelter boy ; karyotyping showed female karyotype with mosaicism for two marker chromosomes 48,XX,+mar1,+mar2[68]/ 47,XX,+mar1[19]/47,XX,+mar2[6]/46,XX[8];  the larger supernumerary marker chromosome (SMC) was characterized as a ring Y-chromosome. Detection of the SRY-region explained the male phenotype.  The smaller second marker chromosome contained the pericentromeric region of chromosome 8. | Isolated  Nonverbal abilities and memory were normal | yes | yes |
| Ross et al,  2008 | Case series  N=50 | Expand the description of the cognitive development phenotype in boys with Klinefelter syndrome (47,XXY). | 47,XXY | Yes, IQ within normal range | yes | yes |
| Ross et al, 2009 | Case control  114 participants, 36 controls | To contrast the cognitive phenotypes in boys with 47,XYY (XYY) karyotype and boys with 47,XXY karyotype [Klinefelter syndrome, (KS)], who share an extra copy of the X-Y pseudo-autosomal region but differ in their dosage of strictly sex-linked genes | 47,XYY (XYY) karyotype and boys with 47,XXY | Isolated, however scores on the DAS GCA suggest that general cognitive ability is mildly diminished in both groups, compared to controls. | yes | yes |
| Bishop et al, 2011 | Case control  135 cases; 68 controls | Speech evaluation and diagnostics for autism | Patients with sex chromosomal anomalies | Isolated and non-isolated  Cases with ASD included (11%) | yes | yes |
| Bishop et al,  2011 | Case control  68 cases | To compare the phenotype in Klinefelter syndrome (KS) with (i) specific language impairment (SLI) and (ii) XXX and XYY trisomies. | Patients with sex chromosomal anomalies | Isolated and non-isolated  Cases with ASD included (11%) | yes | yes |
| Lee et al, 2012 | Case control  110 cases, 52 controls | Examined different language domains and social functioning in the same sample of children with tri-, tetra-, and pentasomy X/Y-aneuploidy. | Patients with sex chromosomal anomalies | isolated and non-isolated  Cases with lower IQ and autistic behavior included | yes | yes |
| Simpson et al,  2014 | Prevalence study  1249 participants: Language impaired 87 probands, 165 siblings, SLI 209 probands, 187 siblings, dyslexia 314, controls 287 | Prevalence of chromosomal anomalies | Patients with language impairment | Isolated | Yes - Karyotyping was only performed in the language-impaired cohort.  Karyotype analysis was performed for all referrals as part of the clinical assessment, regardless of final diagnosis | Yes  Criteria for inclusion in SLIC were language skills more than 1.5SD below that expected for their age on the Clinical Evaluation of Language Fundamentals expressive or receptive language scales; and a non-verbal IQ above 80; and no other obvious explanation for the impaired language, such as an ASD. T |
| Akcan et al, 2018 | Retrospective  case series  N= 23 | Broad phenotype evaluation | 47,XXY | Isolated and non-isolated  Most frequent clinical findings were neurocognitive disorders, speech impairment, social and behavioral problems | yes | no |
| Udhnani et al,  2018 | Case-control  79 participants, 42 controls | To characterize phonemic and semantic verbal fluency in sex chromosome aneuploidies, focusing on sex chromosome tetrasomies and pentasomies | 47, XYY (11), 47,XXX (27), 47,XXY (19), 48,XXXX (1), 48,XXXY (4), 49,XXXXY (7), 48,XXYY (10) | Isolated, however  controls had higher IQ scores than all of the SCA groups. | yes | yes |
| Zampini et al,  2018 | Case- control  15 participants, 15 controls (TD: typically developing) | To describe the first stages of language development in children with SCT in comparison with those in typically developing (TD) children.  To verify the existence of different communicative skills (in both vocal and gestural modality) and to identify the presence of possible early predictors (i.e., low vocabulary size or lack of gesture production) of language impairment in children with SCT. | 47,XXY (n=8)  47,XXX (n=7) | Isolated? | yes | yes |
| Bishop et al., 2019 | Case control  79 cases;  175 controls | Speech evaluation | Patients with sex chromosomal anomalies | Unclear  After excluding children with intellectual disability, autism or hearing problems | yes | yes |
| Matsuzaki et al,  2019 | Case control  N=9 | To investigate whether similar findings are observed in XYY-associated ASD and whether delayed processing is also present in individuals with XYY without ASD | Patients with XYY | Unclear  Participants with nonverbal IQ lower than the 2nd percentile were excluded. | yes | yes |
| Gropman et al,  2020 | Cohort study  N= 67 | To investigate effect treatment on development | Patients with sex chromosomal anomalies | Isolated and non-isolated  Also cases with lower IQ included | yes | yes |
| Van Elst et al,  2020 | Case report  N=1 | To evaluate the scope of language impairments in a patient with Triple X Syndrome | 47, XXX | Isolated and non-isolated  Full-scale IQ (determined with Wechsler Adult Intelligence Scale-III): 70. | yes | yes |
| Capelli et al. 2022 | Case series  N=13 | To describe language development in children with sex chromosome trisomies (SCT) and to test  the predictive value of early language measures on later outcomes. | Thirteen children with SCT were followed longitudinally, diagnosed prenatally | Isolated: linguistic quotient < performance quotient | yes | yes |
| Urbanus et al. 2022 | Case control  N=205 (103 with SCT and 102 without SCT) | To identify language abilities of young children with SCT across multiple language domains and  to identify the percentage of children that, according to clinical  guidelines, have language difficulties | Children with XXX (32), XXY (49), XYY (22), XX (58), XY (44) | Isolated | yes | yes |
| Zampini et al. 2022 | Case controlN=76 (38 with SCTs, 38 TD | To verify the impact of having a diagnosis of SCTs on language development during the second year of life. | XXX (14), XXY (12), XYY (12), XX (15), 23 (XY) | ? | yes | yes |
| ***Other genetic anomalies*** |  |  |  |  |  |  |
| **Chromosomal deletion/ translocation/ inversion** |  |  |  |  |  |  |
| Thompson et al,  1985 | Case series  N=3 | To study 18p- phenotype | 3 children  2,5-9 yrs | Isolated? And non-isolated  Case 1: PIQ 90  Case 2: IQ 64 nonverbal test  Case 3: IQ 69? Borderline? | yes | yes |
| Bogart et al, 1986 | Case report | Prenatal diagnosis and follow up of a child with a complex chromosome rearrangement | karyotype is 46,XX,t(6;11)(p21;q21),t(11;21) (q21;p13),inv(6)(p21q11) | Isolated  Normal development except for speech at 2.5 years of age | yes | yes |
| Weistuch et al,  1996 | Case report | Description of specific expressive language impairment with verbal apraxia in chromosomal translocation | M  4;8 years  de novo  (karotype: 46, XY, t(1p22; 2q31}). | Isolated | yes | yes |
| Kwasnicka et al,  2005 | Case report  + cohort | To unravel the impact of inversion on transporter ATPase gene (ATP13A4) as a cause of language delay | Subject with language delay  [46XX, inv(3)(q25.32 – q29) | Isolated | yes | yes |
| Moralli et al, 2015 | Case report  N=1 | To describe language impairment in a case of a complex chromosomal rearrangement with a breakpoint downstream of FOXP2 | Young female – follow up 16 yrs | Isolated  At 10;8 years her total IQ score was in the normal-low range (88), her verbal IQ was low (74) while her nonverbal IQ was above the mean for her age (106). | yes | yes |
| Lai et al, 2000 (See also Lai et al 2001) | Case series | To unravel region on 7q31 for a candidate gene for DLD | Three-generation pedigree with DLD transmitted as AD trait by a chromosomal translocation  + 2 cases with chromosomal translocation in the same region | Isolated | yes | yes  DLD and verbal dyspraxia |
| Tomblin et al, 2009 | Case series | To study language characteristics FOXP2 | Family  T:18-20 yrs; B: 50-52 yrs  Chromosome 7;13 translocation involving FOXP2 | Isolated  Both families show below average scores and both families show a pattern where the average verbal score is 10 points below the average performance IQ. | yes | yes |
| **CNV anomaly** |  |  |  |  |  |  |
| Chui et al, 2011 | Case report | To describe 7p22.1 microduplication detected by array CGH as a probable cause of DLD | Case with 7p22.1 dup | Isolated | yes | yes |
| Ceroni et al,  2014 - | Case report | To explore role ZNF277 as a cause of SLI | Homozygous microdeletion of exon 5 in ZNF277 in a girl with specific language impairment | Isolated | yes | yes |
| Centanni et al,  2015 | Case series  N=8 | To explore role of CNV’s as cause of DLD | All 4 cases:  large gains in an overlapping region at 15q11.2.  in addition: pt 1: 13q21.1 (gain) en 12p13.33 (loss); pt 2: 10q21.1 (loss) en 16p11.2 (loss); pt 3: 9p24.3 (loss) en 22q13.33 (gain); pt 4: 7q11.23 (gain) | Isolated,  All participants were required to have normal cognition based on a standard score of 75 or higher on the RIAS. | yes | yes |
| Pettigrew et al, 2015 | cohort – n=85 followed by case report  N=1 | Identification of a specific CNV as a cause for DLD | Girl; <7;8 years | Isolated  No evidence of intellectual disability but was notably lower at age 7.8 years than earlier in development. | yes | yes |
| Rakonjac et al,  2015 | Case control  N=11 both groups | Comparing cases with 22q11.2 (group E1) and 22q11.2 like phenotype without a deletion (group E2) and controls (C) | 5-10 years | Isolated and non-isolated  Children from groups E1 and E2 achieved lower scores compared to children from group C | yes | yes |
| Ricardi et al, 2015 | Case report  (family) | Unraveling contribution of cryptic 13q34 and 4q35.2 deletions in an Italian family with DLD | Proband: 13 yrs; sister: 6 yrs  2 sibs:  Sib 1) dupXp22.11(22,702,464–22,816,629) del4q35.2(190,864,518–191,133,858) del13q34(111,915,389–113,104,705)  Also pathogenic variant in PTPN11 | Isolated | yes | yes |
| Kalnak et al,  2018 | Case control  58 probands; 159 family members; 76 controls | Evaluating CNV’s as cause for DLD | Cases with language impairment  16p11.2 (2x proband, 1x sibling); 17q12 (1x proband); 18p11.32-p11.22 (1x sibling); 47, XXY (1x proband), Xp22.31-Xp22.33 (1x sibling) | Isolated | yes | yes |
| **Gene Variant** |  |  |  |  |  |  |
| Lai et al, 2001  (See also Lai et al 2000) | Family study | Identifying causative gene | Same family (KE) as in Lai et al 2000 |  |  |  |
| Unger et al, 2007 | Case report | FLNA as a cause for FG syndrome | M  18 months | Isolated?  At 18 months – development generally good – however speech delay | yes | no |
| Addis et al,  2010 - | Case control  13 family (8 affected, 5 unaffected); 34 controls (age matched) | Identification underlying genetic cause | AD family; Three-generation German family who present with an apparently simple segregation of language impairment.  Candidate gene sequencing:  CNTN1, FOXJ2, GRIN2B, NELL2, NAB2 and SRGAP1. | Isolated  Normal IQ | yes | yes  ERP experiment: phonological discrimination  Behavioral testing: NWR test |
| Chen et al,  2017 - | Prevalence study  N=43 | Gene variants identified by WES | Cases with language impairment | IQ? | yes | yes |
| Andres et al. 2021 | Case control  N=11 (family with SLI) and n=175 (unrelated individuals with SLI) | To better understand the genetic architecture of SLI | Family with SLI and control group of unrelated individuals with SLI | Isolated | yes | Not mentioned in the article which tests, but subjects were recruited from Language Acquisition Studies Lab at KU |
| **Metabolic disorder** |  |  |  |  |  |  |
| IJlst et al,  2002 | Case report | 3-Methylglutaconic aciduria type I is caused by mutations in AUH | 2 cases from 8 previous reported cases this metabolic disorder; Mutation analysis of AUH in two patients revealed a nonsense mutation (R197X) and a splice-site mutation (IVS8-1GrA), demonstrating that mutations in AUH cause 3-methylglutaconic aciduria type I | Isolated | yes | not clear |
| Yeung et al,  2013 | Case report | Phenotype evaluation in a case with dihydropyrimidinase deficiency | M  2,8 years | Not mentioned  Motor development was appropriate for his age. Socially, the child showed poor eye contact, but no restriction of other interests or ritualistic behavior | yes | yes |
